# Psychopathological symptoms in school-aged children after a traumatic event

**DOI:** 10.1186/s13034-025-00869-6

**Published:** 2025-02-21

**Authors:** K. S. Plümacher, J. K. Loy, S. Bender, M. Krischer

**Affiliations:** Department of Child and Adolescent Psychiatry, Psychosomatics, and Psychotherapy, University of Cologne, Faculty of Medicine and University Hospital Cologne, Cologne, Germany

## Abstract

**Background:**

Traumatic experiences in childhood can have far-reaching and serious consequences for the development of those affected. Little is known about the age- and sex-specific symptom patterns in children and adolescents following a traumatic event. These do not always manifest in symptoms that meet the diagnostic criteria for post-traumatic stress disorder according to ICD 10.

**Methods:**

In an outpatient cohort of 84 children and adolescents aged 6 to 18 years, we utilised the dimensional Child Behaviour Checklist (CBCL) to analyse symptoms occurring within twelve months of a traumatic event as defined in ICD 10. Regression models were applied to examine the effects of sex, age, and their interaction. CBCL (sub-)scales from caregivers served as the outcome variables.

**Results:**

The most severe symptoms were externalising symptoms found in boys aged six to below twelve years, while girls of the same age showed the fewest symptoms. No fully consistent picture regarding age- and sex-effects was found across the (sub-)scales, however, the most consistent finding for all scales was an interaction between age and sex, resulting in a convergence between boys and girls with age. Adolescent males and females were affected to a similar extent.

**Conclusion:**

Knowledge of age- and sex-specific patterns in children and adolescents following a traumatic event is essential to recognise possibly trauma-related symptoms at an early stage, initiate adequate treatment. Considering that trauma can exacerbate or complicate externalising symptoms, and vice versa, it is crucial to integrate trauma-specific interventions into the treatment plan for those affected. Developing comprehensive, age- and sex-specific diagnostic criteria for trauma-related disorders might not only improve early detection and treatment but also ensure that interventions address both emotional and behavioural dimensions effectively. Future research should focus on refining these criteria and exploring the interplay between trauma and co-occurring conditions to enhance treatment outcomes.

**Supplementary Information:**

The online version contains supplementary material available at 10.1186/s13034-025-00869-6.

## Introduction

Childhood trauma can have profound and lasting effects for affected individuals with far reaching consequences for psychological, emotional, and social development, recently highlighted by studies on adverse childhood experiences [11, 14]. Adults affected by traumatic experiences in childhood, showed higher rates of substance abuse, depression, ADHD, psychotic experiences, bipolar disorders and suicide attempts as well as an increased prevalence of stroke, myocardial infarction, coronary heart diseases and diabetes [4, 16, 21, 27]. Fergusson and colleagues [10] showed an increased risk of developing mental health problems in children after sexual or physical abuse. Klicken oder tippen Sie hier, um Text einzugeben.

The most common secondary diagnoses that occur in children with post-traumatic stress disorder are depression, anxiety and hyperactivity and attention disorder [5]. Trauma-related disorders could manifest rather as a variety of psychiatric or behavioural symptoms and a wide range of symptoms, like the various clinical manifestations of intrusions, making the diagnostics of a post-traumatic stress disorder (PTSD) difficult [26]. Additionally, a traumatic event does not always lead to symptoms that meet the criteria for PTSD or fully satisfy the diagnostic criteria for post-traumatic stress disorder according to DSM 5 or ICD 10. Instead, other, less typical symptoms may emerge that are not commonly recognised as classic responses to traumatic experiences. Therefore, it is crucial to move beyond a strict focus on diagnosis and adopt a more comprehensive and holistic approach to understanding how affected individuals manifest their reactions, particularly in children and adolescents, where responses to trauma may present differently. Conversely, psychological symptoms or disorders are inherently multifactorial, and the presence of trauma does not necessarily imply a direct causal link to trauma-related symptomatology. Research from the E-Risk Study provides crucial insights into this complexity. For example, Lewis et al. [18, 19] found that trauma exposure is associated with an increased risk for diverse psychiatric outcomes, but trauma-exposed young people frequently had pre-existing vulnerabilities that complicated this relationship. Further, in examining complex trauma specifically, Lewis et al. [19] demonstrated that individuals exposed to repeated interpersonal trauma showed more severe psychopathology and cognitive deficits, yet early childhood vulnerabilities—such as internalising and externalising symptoms, cognitive function, and socioeconomic factors—played a substantial role in moderating these associations. The findings have direct clinical implications: when adolescents present with psychological difficulties following complex trauma, these might reflect manifestations of pre-existing vulnerabilities rather than—or in addition to—trauma-related disorders. Careful differential diagnosis is essential. Otherwise, trauma symptoms can be falsely attributed to other diseases, which has a major impact on the choice of treatment as therapeutic approaches may vary substantially.

Type and severity of trauma symptoms were shown to vary by sex and age. Current evidence indicates that women experience PTSD more frequently and with greater severity, including higher rates of comorbidity, compared to men ([7, 39]). Haag and colleagues [12] examined children and adolescence, who reported on having experienced a traumatic event. The number of PTSD diagnosis at age of eight and ten was similar in boys and girls, at age 13 girls appeared to be significantly more likely affected by PTSD. These differences are based on the cognitive and brain structure development, of hormonal and epigenetic influences as well as on social role models [7]. Similarly, population-based research has shown that females are generally more prone to depression and anxiety, while males are more likely to develop attention-deficit and conduct disorders [23]. In high-risk populations, boys are more frequently diagnosed with ADHD, whereas girls tend to be diagnosed with depression [23]. Age also plays a role, with ADHD prevalence higher in children, while adolescents show increased rates of affective, conduct, and anxiety disorders. Hagan and colleagues [13], for instance, found that trauma-exposed girls between the ages of three to six years more often expressed PTSD and dissociation symptoms compared to boys, as reported by their parents. Together, these findings underscore the complex interplay of age and sex in the development and expression of psychiatric disorders and trauma-related symptoms.

Several studies further highlight symptom differences between younger children compared to older children and adolescence after traumatisation [13, 17]. There is increasing evidence that the nature of the expression of trauma symptoms depends on the emotional and cognitive stage of development. In young children, for example, hyperarousal manifests itself in the form of separation anxiety, in adolescents in the form of risk-taking behaviour. Despite these differences, both traumatised children and adolescents share common frequent symptoms, such as emotional and cognitive difficulties. Symptoms can include anxiety, PTSD, dissociative behaviours, and challenges in emotional regulation [17]. These findings regarding the effects of sex and age are highly relevant, but the interaction between these factors remains insufficiently understood. We believe that identifying sex- and age-specific symptom patterns is crucial for accurate classification and optimal treatment decisions. This study addresses this critical, yet underexplored, aspect of trauma research by examining how sex and age interact to influence symptom expression. By offering this novel perspective, our research contributes to a more nuanced understanding of trauma's developmental trajectory and its implications for personalised treatment strategies.

Given the limited literature to support the formulation of specific hypotheses, the study adopts an exploratory approach. While we hypothesise potential effects of sex and age, as well as their interaction, these relationships have not been clearly established in the context of our study. Therefore, our analysis will approach these factors in a data-driven manner, without predefined directional hypotheses. This paper aims to: (1) investigate the interactions between sex and age in traumatised children across all scales and subscales of the CBCL, and (2) explore sex-specific models to examine age effects in the assessment of trauma-related symptoms using the CBCL in traumatised children.

## Methods

### Study design

For the present study, data were drawn from the prospective research project titled Consequences of Trauma and Trauma-Related Disorders in Children and Adolescents—A Study at the Cologne Trauma Outpatient Clinic for Children and Adolescents at the University of Cologne. Participants were recruited between 2010 and 2012 from the trauma outpatient service of the child and adolescent psychiatric department at the University Hospital Cologne. This study was approved by the ethics committee of the University of Cologne (number: W-10-2-006). All participants provided informed consent.

The focus of the current study was not solely on the presence or absence of a formal PTSD diagnosis. Instead, our aim was to investigate the range and severity of trauma-related reactions in children and adolescents. Diagnoses of PTSD and other trauma-related disorders were made by experienced clinicians according to ICD 10 criteria via clinical interviews with children as well as their parents ensuring a high level of diagnostic accuracy. We sought to understand how different age groups and sexes express symptoms following trauma, irrespective of whether they meet the criteria for a formal PTSD diagnosis. We focused on school-aged children and adolescents (ages 6–18) to ensure developmental comparability and capture trauma symptom patterns during key developmental stages, recognising the distinct neurological and emotional processing of traumatic experiences during this period. A separate analysis of children under 6 years was not feasible due to insufficient case numbers. No strict inclusion or exclusion criteria for the study were applied except from age: all patients who presented at the Trauma Outpatient Clinic with any trauma-related disorder were included, provided they were above the age of 6 and under the age of 18 and had experienced trauma within the past twelve months. This approach allowed for a broader exploration of trauma reactions beyond the constraints of traditional PTSD diagnostic categories.

### Measures

Age groups: Due to the sample size and in order to obtain reliable results, two age groups were examined: children (6–11 years) and adolescents (12–18 years). This classification was made analogous to the age grouping for the standardization of the Child Behaviour Checklist (CBCL/6-18R, hereinafter referred to as CBCL) [26].

The type of trauma was assessed via interviewing. A binary variable distinguishing between personal and non-personal trauma was created and included in the analyses. Personal trauma includes among others sexual or physical abuse, death of close family members, and neglect; non-personal trauma includes accidents and natural disasters. However, only in some cases, type of trauma was explicitly specified, often solely the distinction between personal and non-personal trauma was made.

The CBCL is a widely used cost- and time-efficient tool with high sensitivity [2, 31]. It assesses a variety of behavioural and emotional problems and somatic complaints. Diagnostic criteria of PTSD in children and adolescents are based on the ICD 10 and include intrusion symptoms like re-experiencing of the traumatic event, avoidance strategies and negative alterations in cognitions and mood in association with the traumatic event and increased physiological arousal. In our trauma outpatient clinic, the CBCL is routinely administered to all patients at their initial presentation as a general screening tool, regardless of study enrolment. This approach helps in the early identification of behavioural issues related to trauma exposure. The CBCL for ages 6–18 consists of 113 questions regarding behaviour problems to be completed by parents or other direct caregivers in case of professional care by choosing between 0 (not at all), 1 (sometimes), or 2 (all the time). It is subdivided into three main scales (total score, external and internal) and nine subscales (dissocial behaviour, social withdrawal, somatic complaints, anxious/depressive, social problems, schizoid/obsessive, other problems, attention problems), each of which served as a dependent variable for examining the children’s problem behaviour.

### Statistical analyses

Preceding the statistical models, descriptive analyses with the raw scores were conducted in a first step. Means and standard deviation for all scales and subscales, categorised by sex and age group were calculated. The cut-offs derived from the German normative sample. They are employed to categorise children/adolescents separately for girls and boys from our sample into normal or at risk/problematic. The determination of these cut-offs in the norm-sample involved specifying that the top 15% of respondents should fall within the at risk range, while the top 8% should be in the conspicuous range for the main scales. Similarly, for the subscales, the specified percentages in the norm-sample were 5% and 2%, respectively [33]. This forms the basis for comparing the proportions of children/adolescents in our sample who are to be considered at risk/problematic.

This was followed by the examination of sex and age effects on CBCL scores. Multiple regression models were calculated, one for each scale/subscale of the CBCL. The models included the dichotomous variable sex and age group as well as the interaction between sex and age group. Female children served as reference category. Second, the models were calculated for boys and girls separately. Conducting subgroup analyses for boys and girls aims to unveil potential nuances in how age impacts the outcome within each sex.

Following a stepwise regression (forward selection), the models were calculated with and without the trauma type covariate. Its inclusion was deemed meaningful, contributing to an increased R^2^ value, reflecting specific patterns or influences that may be better captured, even though not meeting the traditional statistical significance threshold, causing that all models were adjusted for the covariate trauma type distinguishing between personal versus non-personal trauma, to control for its potential impact on the CBCL scores. Graphical representations of the interaction models were generated using the “margins” and “marginsplot” commands in Stata, aiding in the visualization and interpretation of our findings. Pearson’s chi-square was applied to test whether there was a difference in trauma type between the sexes. Alpha was set at 0.05 and Bonferroni adjustment to account for multiple testing. All statistical analyses were conducted using Stata Stata/SE 18 (Stata Corp LP; College Station, TX, USA).

## Results

### Descriptive statistics

Descriptive statistics are summarised in Table 1. Of *n* = 130 patients attending the trauma outpatient service who agreed to participate in the study, CBCL data were available for *n* = 93. For the others, either no CBCL was available at all or it was too incomplete to be used. After exclusion of *n* = 9 children below the age of six years, the total sample size comprised *n* = 84. Among these participants, *n* = 55 were female, *n* = 41 were in the child age group. The mean age ± standard deviation (SD) in this subsample was 11.4 ± 3.6 years. A personal trauma had been experienced by *n* = 66, a non-personal by *n* = 12 study participants.

**Table 1:** Overview of demographic characteristics and type of trauma

	N	%
Sex		
Female	55	65.48
Male	29	34.52
Nationality		
German	72	85.71
Non-German	10	11.90
Missing	2	2.38
Age group		
Children 6–11 years	41	48.81
Female		
23 (27.4%)		
Male		
18 (21.4%)		
Teens 12–18 years	43	51.19
Female		
32 (38.1%)		
Male		
11 (13.1%)		
Trauma type		
Personal	72	85.71
Non-personal	12	14.29

Cronbach's Alpha for the total score of the CBCL was 0.86, indicating good internal consistency. For the subscales, the internal consistency was as follows: internalising problems α = 0.79, externalising problems α = 0.87, dissocial behaviour α = 0.60 and aggressive behaviour α = 0.82. Table 2 presents an overview of means for all scales and subscales, categorised by sex and age group. Among females, children showed a lower mean total score (33.91) compared to teenagers (43.00). For males, this trend was observed on the internalising scale, where children had a lower mean score (12.61) than teenagers (18.18). However, for the total score among males, children had a higher mean score (20.22) than teenagers (11.73), showing an opposite trend compared to females. This same pattern was seen on other scales, such as the external scale, with mean scores of 8.26 and 12.69 for females, and 20.22 and 11.73 for males, respectively.

**Table 2:** Overview of means and standard deviation for all scales and subscales, categorised by sex and age group

Variable	Children female			Teens female			Children male			Teens male		
	n	Mean	Std. Dev	n	Mean	Std. Dev	n	Mean	Std. Dev	n	Mean	Std. Dev
Total	23	33.91	21.45	32	43.00	24.55	18	54.17	31.42	11	45.09	24.83
Internal	23	11.17	7.76	32	15.88	9.78	18	12.61	9.04	11	18.18	8.41
External	23	8.26	7.60	32	12.69	10.28	18	20.22	11.95	11	11.73	11.33
Social withdrawal	23	3.26	2.45	32	5.06	2.95	18	3.89	2.95	11	7.27	4.08
Somatic complaints	23	2.09	2.87	32	3.41	3.31	18	1.39	2.00	11	3.82	3.92
Anxious, depressive	23	6.39	4.09	32	8.22	5.66	18	8.17	6.39	11	8.36	4.11
Social problems	23	2.30	1.96	32	1.97	2.10	18	4.11	3.32	11	2.27	2.53
Schizoid, obsessive	23	1.61	1.64	32	1.38	1.54	18	2.06	2.39	11	2.73	2.72
Dissocial behaviour	23	1.48	2.04	32	4.13	4.40	18	4.56	4.30	11	3.91	4.28
Aggressive behaviour	23	6.78	5.88	32	8.56	6.39	18	15.67	8.53	11	7.82	7.70
Other problems	23	7.43	5.81	32	7.59	4.35	18	10.28	7.08	11	6.91	5.19
Attention problems	23	3.52	2.83	32	5.00	3.52	18	7.00	4.42	11	7.09	4.61

Table 3 provides an overview of the proportions of participants with problem CBCL scores on the main scales and selected subscales. In comparison to the norm sample, where 23% on the main scales and 7% on the subscales are classified as at risk or problematic, our sample shows significantly higher proportions. This is especially pronounced on the internalising scale, with high rates observed across both age groups for boys (77.8% and 81.8%) and girls (65.2% and 78.1%). Elevated proportions are also evident for the total score, the external scale, and aggressive behaviour among younger boys, with 72.2%, 77.8%, and 77.8%, respectively.

**Table 3:** Proportion of participants with at risk or problematic CBCL scores in the main and in selected subscales

	Cut-off for at risk CBCL	At risk or problematic CBCL score % (n)	Total n
Total			
Children male	32	72.2% (13)	18
Children female	29	52.2% (12)	23
Teens male	31	72.7% (8)	11
Teens female	29	65.6% (21)	32
External			
Children male	12	77.8% (14)	18
Children female	9	34.8% (8)	23
Teens male	13	45.4% (5)	11
Teens female	10	46.8% (15)	32
Internal			
Children male	7	77.8% (14)	18
Children female	8	65.2% (15)	23
Teens male	8	81.8% (9)	11
Teens female	9	78.1% (25)	32
Aggressive behaviour			
Children male	12	77.8% (14)	18
Children female	10	30.4% (7)	23
Teens male	12	45.4% (5)	11
Teens female	10	34.4% (11)	32
Dissocial behaviour			
Children male	6	27.8% (5)	18
Children female	5	13.0% (3)	23
Teens male	7	18.1% (2)	11
Teens female	6	28.1% (9)	32

### Differences and similarities between boys and girls

Results of the full model (interaction model) are depicted in Table 4. For externalising symptoms and for the subscale AGGRESSIVE BEHAVIOUR, the main effects of sex were statistically significant (β = 16.10; *p* < 0.001 and *β* = 11.87; *p* < 0.001, respectively) while they were not for age group. For these scales, statistically significant interactions between sex and age were found (*β* = − 16.16; *p* = 0.001 and *β* = − 12.11; *p* < 0.001, respectively), indicating higher values for male boys compared to girls and compared to male teenagers. Given the small sample size and the exploratory nature of the analysis, results that are significant without Bonferroni correction are also reported. This includes the interaction effect for total score (β = − 28.37, *p* = 0.024) and dissocial behaviour (*β* = − 4.05, *p* = 0.038).

**Table 4:** Interaction sex*age for all scales and subscales of the CBCL

Variable	Sex			Age			Interaction		
	Coefficient	p	CI	Coefficient	p	CI	Coefficient	p	CI
Total score	**31.014**	0.000	(14.140; 47.888)	12.259	0.081	(− 1.553; 26.071)	− *28.368*	0.024	(− 52.875; − 3.861)
External	**16.095**	0.000	(9.446; 22.744)	5.782	0.038	(0.339; 11.224)	− **16.158**	0.001	(− 25.815; − 6.502)
Internalising	4.162	0.182	(− 1.992; 10.315)	5.269	0.041	(0.232; 10.305)	− 2.520	0.576	(− 11.457; 6.417)
Aggressive behaviour	**11.865**	0.000	(7.394; 16.335)	2.802	0.131	(− 0.858; 6.461)	− **12.109**	0.000	(− 18.602; − 5.616)
Dissocial behaviour	4.231	0.002	(1.597; 6.864)	2.980	0.007	(0.824; 5.136)	− *4.049*	0.038	(− 7.874; − 0.224)
Social withdrawal	1.336	0.202	(− 0.730; 3.402)	1.950	0.024	(0.259; 3.642)	1.110	0.463	(− 1.891; 4.111)
Somatic complaints	− 0.186	0.858	(− 2.256; 1.883)	1.548	0.073	(− 0.146; 3.242)	− 0.120	0.937	(− 3.126; 2.886)
Anxious, depressive	3.386	0.066	(− 0.230; 7.001)	2.051	0.171	(− 0.908; 5.010)	− 3.495	0.189	(− 8.746; 1.755)
Social problems	*2.494*	0.004	(0.837; 4.151)	− 0.184	0.788	(− 1.540; 1.172)	− 1.961	0.109	(− 4.368; 0.445)
Schizoid, obsessive	0.943	0.168	(− 0.407; 2.294)	− 0.226	0.685	(− 1.331; 0.879)	0.585	0.554	(− 1.375; 2.546)
Other problems	*4.689*	0.014	(0.961; 8.416)	0.733	0.633	(− 2.318; 3.784)	− 5.682	0.040	(− 11.096; − 0.268)
Attention problems	4.726	0.000	(2.259; 7.194)	2.070	0.045	(0.050; 4.090)	− 2.223	0.220	(− 5.807; 1.360)

Significant results after Bonferroni correction for 36 comparisons (alpha = 0.0014) are shown in bold. Significant results without Bonferroni correction are shown in italics. Female children served as reference category

**Fig. 1:**
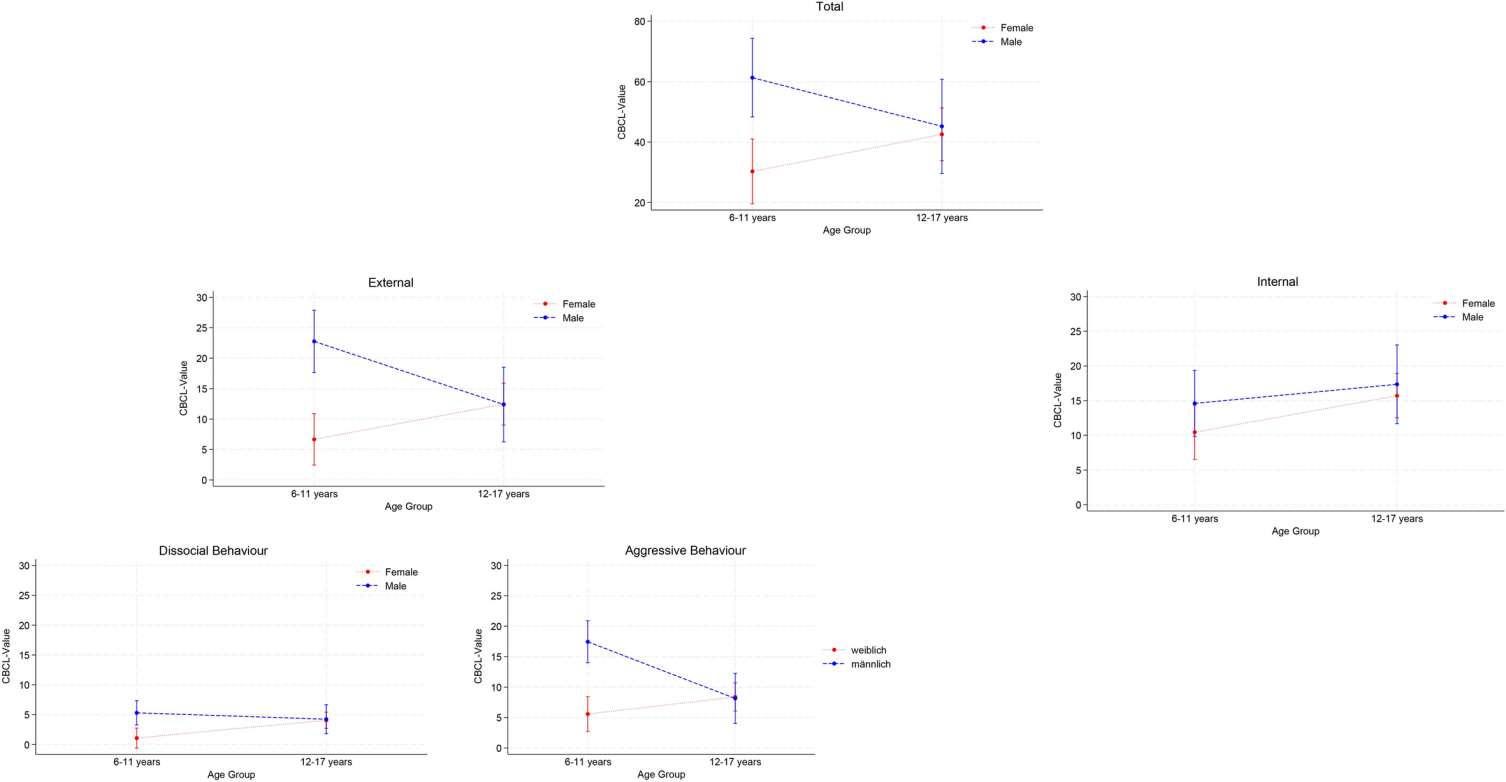
Interaction between sex and age (both dichotomous) for main and subscales

To enhance visualisation, we graphically represented the four scales with statistically significant interaction effects in Fig. 1, illustrating the convergence of values for girls and boys with increasing age. For completeness, the internalising symptoms scale, despite not showing a statistically significant interaction effect, was also included for comparison. Sex-specific models, as detailed in Tables S1 and S2, confirm that there are only minimal statistically significant age effects for both sexes. These models help to identify and confirm the general pattern of female children showing less problematic behaviour than female teenagers while the opposite is observed for males. The sex-specific models provide a more nuanced understanding of age effects and allow a simplified interpretation of individual effects compared to interaction effects.

### Effects of category/type of trauma

Among the participants, 49 females and 23 males had experienced personal trauma, while six female and six male participants reported non-personal trauma. For the remaining six participants, no data was available on trauma group. The Pearson chi-squared analysis indicated that there was no statistically significant difference in trauma type exposure between sexes (*χ*^*2*^(1) = 2.0978, *p* = 0.148). However, it is worth noting that the decision to include trauma type as a covariate in our analysis was based on a stepwise regression (forward selection). In other words, the models were calculated with and without the trauma type covariate. Its inclusion was deemed meaningful, contributing to an increased R^2^ value, reflecting specific patterns or influences that may be better captured, even though not meeting the traditional statistical significance threshold.

## Discussion

Using data from a prospective study of a population of minors requiring treatment, we analysed sex- and age-specific differences in the severity of symptoms following acute trauma within one year in a cohort seeking outpatient treatment. The analysis of the CBCL in children and adolescents aged six to 18 years revealed that the severity and type of symptoms following a traumatic event are influenced by an interaction between sex and age. A major difference between our study and some studies discussed below (e.g., [12, 15]) is the study design. Unlike these longitudinal studies, our cross-sectional approach captures only a single time point. This limits our ability to infer causal relationships or observe changes in symptoms over time, so comparisons with longitudinal findings should be interpreted cautiously and seen primarily as contextual background for our results. The highest symptom severity was found in boys below the age of twelve in the CBCL total score and in the external scale, and its subscales aggressive and dissocial behaviour. In contrast, younger girls appeared to be the least affected among the four groups compared. We observed that in adolescence, girls from the age of twelve years showed higher symptom severity than younger girls, whereas older boys exhibited fewer symptoms compared to younger boys. These results suggest that, according to the CBCL, boys of primary school age tend to show the strongest symptoms following a traumatic event. In adolescence, the characteristics and severity of symptoms become more similar between sexes (see Fig. 1).

### Comparison with existing literature

In our study, we found that the extent of psychological symptoms following a traumatic event is similar in boys and girls during adolescence, whereas other studies report higher rates in females compared to men ([7, 39]). A major difference between our study and the cited studies is the choice of test procedures. We used the CBCL as a screening tool to assess a broad spectrum of psychological problems rather than focusing solely on typical PTSD symptoms. This approach provides a more comprehensive view of the overall burden on children and adolescents, while our results may be less specific to direct trauma effects. Our data showed a high symptom severity in younger boys. This includes ADHD symptoms, which is particularly common in younger boys, regardless of trauma. Since our analyses were not limited to trauma-specific symptoms, we assume that the general distribution of psychiatric conditions influenced our results, possibly explaining the more pronounced symptoms, including hyperactivity, in boys of primary school age compared to PTSD-focused studies. The different selection of test procedures could account for the variations in symptom expression observed between adolescent males and females. Furthermore, the findings should be interpreted in the context of the study population, which consists of children and adolescents seeking outpatient treatment following trauma. The patterns observed may, therefore, overrepresent those with more severe symptoms or functional impairments significant enough to warrant professional help.

### Externalising symptoms in boys: age differences and parental perception

Notably, boys up to the age of twelve showed the strongest manifestation of symptoms in most scales. Externalising behaviours, such as aggressive or dissocial behaviour, were more pronounced in younger boys compared to teenaged boys and compared to both age groups in females. Similarly, Hiscox and colleagues [15] showed that younger boys, defined as below the age of nine years, are strongly affected by traumatisation. We observed fewer and less severe symptoms after trauma in teenage boys compared to younger boys. This raises the question of whether this reflects a true lower burden or if current questionnaires fail to capture these symptoms. If teenage boys exhibit more externalising behaviours, as seen in ADHD, these may shift from hyperactivity to concentration issues, delinquency, or substance abuse [38]. Such symptoms might be less noticeable to parents, as they often appear in peer settings and may only be seen as pathological when clearly pronounced [32]. This suggests a potential underreporting of trauma-related symptoms in male adolescents.

### Trauma recovery in girls: delayed symptoms

In line with our results of young girls showing less severe symptoms than young boys, Hiscox and colleagues found evidence that younger girls demonstrated greater symptom improvement six months after traumatisation than boys of the same age [15]. This more rapid recovery could explain the relatively low level of immediate symptoms observed in young girls. Another possibility is that girls may experience a delayed onset of symptoms after traumatic experiences. Such delayed symptom emergence has been linked to higher rates of mental health conditions in adulthood, including depression, anxiety disorders or eating disorders [25]. This delayed pattern suggests the importance of monitoring girls over time for emerging symptoms, particularly in cases where immediate trauma reactions are minimal.

### Implications for treatment and future research

The interplay between trauma and ADHD complicates both symptom presentation and treatment. Research suggests that co-occurring ADHD and trauma history leads to greater impairment than either condition alone, with adverse childhood experiences potentially exacerbating ADHD symptoms [36].

This highlights the need for diagnostic and treatment approaches addressing both emotional and behavioural aspects. Standard ADHD treatments, including medication, may neglect the emotional impact of trauma and could worsen symptoms like sleep difficulties [38]. Trauma-specific, tailored interventions, incorporating psychoeducation, emotional regulation, and trauma narrative work, are essential. Clinicians should assess, personalise treatment plans, and monitor progress to enhance well-being and treatment outcomes. In the DSM-5 diagnostic criteria for PTSD, externalising behaviour problems are listed exclusively in category E and are described as irritable or aggressive behaviour [22]. This raises the question of whether boys displaying severe trauma through externalising behaviours might be overlooked for a PTSD diagnosis despite potentially benefiting from trauma-specific treatment. The link between childhood trauma and adult ADHD also warrants greater attention, as Peleikis et al. [36] reported that 44% of adult ADHD patients had childhood trauma. Early intervention addressing both trauma and ADHD could improve treatment outcomes. Future studies on intervention effects should explore whether externalising symptoms following a traumatic event respond effectively to established therapies for conduct disorder, or whether trauma-specific factors must be considered in treatment. If the latter holds true, externalising symptoms might reflect an age-dependent psychopathology unique to traumatic experiences, differing from the presentation of PTSD in adults. Conversely, if established therapies suffice, the traumatic event might act as an unspecific stressor that triggers the manifestation of a pre-existing vulnerability. It is likely that both mechanisms coexist, emphasising the need for a personalised approach to therapeutic interventions. More specifically, future studies should evaluate whether a trauma-informed approaches to ADHD therapy is more effective than treating ADHD in a standard way. Here, by'trauma-informed treatment,' we mean approaches that are specifically aware of and responsive to a history of trauma, even if not directly targeting PTSD. This allows for flexible, individualised treatment choices that are sensitive to trauma-related needs, potentially improving outcomes by integrating trauma-informed care into ADHD treatment where relevant. Such an approach may facilitate the initiation of the most beneficial therapy as early as possible, particularly for patients who would otherwise receive ADHD-focused treatment alone. Finally, future studies should investigate whether these are more successful than ADHD therapy alone when a combination of ADHD-typical externalising behaviours and trauma is involved. It can be assumed that the high prevalence of externalising symptoms may be partially biased: Specifically, children exhibiting externalising symptoms as opposed to internalising ones might be overrepresented in trauma outpatient clinics.

This perspective aligns with van der Kolk and colleagues’ [28, 29] influential conceptualisation of developmental trauma disorder (DTD). They argue that the impact of early trauma is often not fully captured by traditional PTSD criteria. The concept of DTD emphasises how chronic childhood trauma can lead to complex patterns of emotional dysregulation, impaired self-perception, and difficulties in interpersonal functioning. These symptoms may not always fit within existing diagnostic categories, potentially leading to misdiagnosis or inadequate treatment approaches. Recognising these broader consequences of early trauma underscores the importance of comprehensive, trauma-informed interventions tailored to the individual needs of affected children and adolescents.

For internalising problems, previous studies [20] found girls to be more affected than boys. However, our data did not show a statistically significant sex difference in internalising problems. Longitudinal studies are necessary to determine if these girls exhibit more internalising behaviour in adulthood compared to boys. This would support the hypothesis of a delayed trauma response in girls. An alternative explanation for the lack of a significant sex difference could be that parents are more attuned to externalising behaviours and thus more likely to seek treatment for these, potentially leading to an underreporting of internalising problems.

While we focused on general psychopathology in this paper, it is important to note that trauma-specific instruments, such as the ETI-KJ, PROPS, and CROPS, were also utilised in our study to assess trauma-related symptoms. Future studies could benefit from incorporating these instruments, along with others like the UCLA PTSD-RI [9] or CRIES [37], to explore the potential parallels and correlations between general and trauma-specific psychopathology. This approach could offer valuable insights into the complex nature of trauma-related mental health outcomes, complementing our findings and further enhancing the understanding of trauma’s impact.

### Holistic assessment

Overall, the findings from this study emphasise the importance of adopting a holistic perspective when assessing trauma responses, particularly in children and adolescents. Moving beyond rigid diagnostic categories to consider the full spectrum of psychological and behavioural manifestations can lead to more effective and personalised treatment approaches. This broader perspective allows for more individualised care, recognising that trauma responses in children and adolescents can be complex and multifaceted, often requiring tailored interventions that address the unique ways in which they process and express their experiences.

## Limitations

When interpreting the results, some limitations have to be considered. First, only a small sample could be included in the study. Nevertheless, statistically significant results were obtained. Second, only parental ratings of the symptoms were examined. There is evidence on differences between (self) and external (caregivers) ratings, especially in adolescents [8]. Therefore, the reliance on caregiver-rated symptoms may potentially impact the validity of our findings due to differing perspectives on behavioural and emotional expressions. This discrepancy could lead to an under- or overestimation of certain symptoms. Third, the cross-sectional design of the study limits possibilities of inferences as no symptom development with increasing age can be assessed. Fourth, due to the small number of patients, age could only be included as a binary variable, and other relevant variables, such as the exact time of the trauma, socio-economic conditions, or previous therapeutic treatments could not be accounted for in our analysis. These factors, along with potential confounding variables such as family history of mental health issues and access to mental health services, may have influenced the observed associations by affecting both risk and resilience factors related to mental health outcomes [3]. Further, it is important to acknowledge that the CBCL provides information on the frequency of symptoms rather than the quality or nature of these symptoms. This limitation means that the reported behaviours may not directly correlate with trauma exposure alone, instead, they could be influenced by various environmental factors or caregiving responses. Consequently, while the CBCL offers valuable insights into symptom prevalence, additional assessment tools might be necessary to capture the complexity and specific qualities of trauma-related symptoms [1]. A limitation of this study is the age of the data, which was collected between 2010 and 2012. However, we consider it unlikely that fundamental age and sex effects have changed significantly over time. Moreover, this study does not aim to provide new norms or develop a new instrument. Finally, variations between boys and girls, as well as between younger and older children exist in norm-samples [30]. However, the current results did not account for these differences in cut-off values derived from normative samples for identifying problem scores on the respective scales and subscales.

## Conclusion

In conclusion, this exploratory cross-sectional study highlights a significant interaction between sex and age in the manifestation of symptoms following a traumatic event in children and adolescents. Our findings indicate that while younger children exhibit statistically significant sex differences in symptom severity, adolescent males and females generally show comparable symptom profiles. This suggests that developmental factors might play a role in how trauma responses are expressed across different age groups.

To gain a more comprehensive understanding of these interactions, further research with longitudinal data is needed. These studies should aim to include younger children, employ a more nuanced approach to age differentiation, and extend the observation period beyond twelve months post-trauma. It is also essential to consider a broader range of outcome variables to capture all potential adverse reactions to trauma.

Our results particularly highlight that primary school-aged boys exhibit more severe symptoms of aggressive and dissocial behaviour compared to older boys and girls. This finding raises concerns about the potential underdiagnosis of PTSD in younger boys, who may not receive optimal treatment due to symptom profiles that do not align with traditional PTSD criteria. Consequently, future studies should assess whether it is appropriate for the development of diagnostic manuals to consider including externalising behavioural patterns in the assessment and treatment of trauma. Such adjustments could enhance the accuracy of PTSD diagnoses and ensure that appropriate treatment options are utilised for all affected individuals.

## Supplementary Information

Supplementary Material 1.

## Data Availability

Data cannot be shared openly to protect study participant privacy.
