# Supplementary material for: Psychopathological symptoms in school-aged children after a traumatic event
Source: Child Adolesc Psychiatry Ment Health. 2025 Feb 21;19:12. doi: 10.1186/s13034-025-00869-6 (PMC11846328; doi:10.1186/s13034-025-00869-6)
Supplement: Supplementary file 1 — Supplementary Material 1. [file 13034_2025_869_MOESM1_ESM.docx]

# Supplementary Material

Table S1 Evaluation of scales and subscales of the CBCL in female subjects.

| Variable |  |  |  |
| --- | --- | --- | --- |
|  | **Coefficient** | **p** | **CI** |
| Total | 12.042 | 0.062 | (-0.619; 24.703) |
| External | 5.739 | 0.023 | (0.829; 10.650) |
| Internal | 5.259 | 0.045 | (0.119; 10.399) |
| Aggressive behaviour | 2.781 | 0.088 | (-0.428; 5.989) |
| Dissocial behaviour | 2.959 | 0.005 | (0.947; 4.970) |
| Social withdrawal | 1.975 | 0.015 | (0.400; 3.549) |
| Somatic complaints | 1.542 | 0.092 | (-0.262; 3.345) |
| Anxious, depressive | 2.022 | 0.167 | (-0.874; 4.918) |
| Social problems | -0.205 | 0.721 | (-1.352; 0.942) |
| Schizoid, obsessive | -0.232 | 0.609 | (-1.137; 0.673) |
| Other problems | 0.631 | 0.647 | (-2.122; 3.384) |
| Attention problems | 2.033 | 0.021 | (0.318; 3.747) |

Table S2 Evaluation of scales and subscales of the CBCL in male subjects.

| Variable |  |  |  |
| --- | --- | --- | --- |
|  | **Coefficient** | **p** | **CI** |
| Total | -14.819 | 0.247 | (-40.666; 11.028) |
| External | -10.124 | 0.057 | (-20.561; 0.313) |
| Internal | 2.806 | 3.794 | (-5.062; 10.674) |
| Aggressive behaviour | -9.183 | 0.015 | (-16.437; -1.928) |
| Dissocial behaviour | -0.942 | 0.626 | (-4.893; 3.010) |
| Social withdrawal | 2.917 | 0.064 | (-0.187; 6.020) |
| Somatic complaints | 1.465 | 0.211 | (-0.891; 3.821) |
| Anxious, depressive | -1.271 | 0.605 | (-6.294; 3.753) |
| Social problems | -2.017 | 0.146 | (-4.793; 0.759) |
| Schizoid, obsessive | 0.395 | 0.729 | (-1.942; 2.733) |
| Other problems | -4.342 | 0.129 | (-10.045; 1.362) |
| Attention problems | 0.068 | 0.973 | (-4.043; 4.178) |
